# *Salmonella* Typhi whole genome sequencing in Rwanda shows a diverse historical population with recent introduction of haplotype H58

**DOI:** 10.1371/journal.pntd.0011285

**Published:** 2023-06-16

**Authors:** Jean Pierre Rutanga, Tessa de Block, Wim L. Cuypers, Josephine Cafmeyer, Marjan Peeters, Esperance Umumararungu, Jean Claude S. Ngabonziza, Aniceth Rucogoza, Olivier Vandenberg, Delphine Martiny, Angélique Dusabe, Théoneste Nkubana, Gordon Dougan, Claude Mambo Muvunyi, Ivan Emil Mwikarago, Jan Jacobs, Stijn Deborggraeve, Sandra Van Puyvelde

**Affiliations:** 1 College of Science and Technology, University of Rwanda, Kigali, Rwanda; 2 Institute of Tropical Medicine, Antwerp, Belgium; 3 Department of Microbiology, Immunology and Transplantation, KU Leuven, Leuven, Belgium; 4 Department of Computer Science, University of Antwerp, Antwerp, Belgium; 5 Rwanda Biomedical Centre, Kigali, Rwanda; 6 Department of Clinical Biology, University of Rwanda, Kigali, Rwanda; 7 Department of Microbiology, Laboratoire Hospitalier Universitaire de Bruxelles (LHUB-ULB), Hôpital Erasme-Cliniques universitaires de Bruxelles, Université Libre de Bruxelles, Brussels, Belgium; 8 Department of Microbiology, Laboratoire des Hôpitaux Universitaires de Bruxelles – Universitaire Laboratorium Brussel (LHUB-ULB), Brussels, Belgium; 9 National Reference Centre for Campylobacter, CHU Saint-Pierre, Brussels, Belgium; 10 Faculté de Médecine et Pharmacie, Université de Mons (UMONS), Mons, Belgium; 11 Centre Hospitalier Universtaire de Kigali (CHUK), Kigali, Rwanda; 12 Department of Medicine, Jeffrey Cheah Biomedical Centre, University of Cambridge, Cambridge, United Kingdom; 13 College of Medicine and Health Sciences, University of Rwanda, Kigali, Rwanda; 14 Wellcome Trust Sanger Institute, Hinxton, United Kingdom; 15 Laboratory of Medical Microbiology, Vaccine & Infectious Disease Institute, University of Antwerp, Antwerp, Belgium; University of Pittsburgh, UNITED STATES

## Abstract

*Salmonella enterica* serovar Typhi (*S*. Typhi) is the cause of typhoid fever, presenting high rates of morbidity and mortality in low- and middle-income countries. The H58 haplotype shows high levels of antimicrobial resistance (AMR) and is the dominant *S*. Typhi haplotype in endemic areas of Asia and East sub-Saharan Africa. The situation in Rwanda is currently unknown and therefore to reveal the genetic diversity and AMR of *S*. Typhi in Rwanda, 25 historical (1984-1985) and 26 recent (2010-2018) isolates from Rwanda were analysed using whole genome sequencing (WGS). WGS was locally implemented using Illumina MiniSeq and web-based analysis tools, thereafter complemented with bioinformatic approaches for more in-depth analyses. Whereas historical *S*. Typhi isolates were found to be fully susceptible to antimicrobials and show a diversity of genotypes, i.e 2.2.2, 2.5, 3.3.1 and 4.1; the recent isolates showed high AMR rates and were predominantly associated with genotype 4.3.1.2 (H58, 22/26; 84,6%), possibly resulting from a single introduction in Rwanda from South Asia before 2010. We identified practical challenges for the use of WGS in endemic regions, including a high cost for shipment of molecular reagents and lack of high-end computational infrastructure for the analyses, but also identified WGS to be feasible in the studied setting and giving opportunity for synergy with other programs.

## Author summary

Typhoid fever, caused by *S*. Typhi, remains a public health threat in low- and middle-income countries in Asia and sub-Saharan Africa. Country data on antimicrobial susceptibility and genetic diversity of circulating *S*. Typhi is crucial for surveillance, treatment guidelines and prevention. Typhoid fever is endemic in Rwanda but there are no data on the diversity of *S*. Typhi genotypes, genetic evolution, or the association with antimicrobial resistance (AMR) in the country. Therefore, we conducted whole genome sequencing (WGS) of 25 *S*. Typhi historical (1984-1985) and 26 recent (2010-2018) isolates from Rwanda. Whereas the historical population is diverse and susceptible to antimicrobials, the recent *S*. Typhi population in Rwanda is dominated by genotype 4.3.1.2 (H58) which is associated with high AMR rates and is dominant in Asia and East Africa. Furthermore, we present a workflow for implementation of WGS to support typhoid surveillance and identified challenges of this workflow in endemic regions.

## Introduction

*Salmonella enterica* subspecies *enterica* serovar Typhi (hereafter *S*. Typhi) is the cause of typhoid fever and has been estimated to be responsible for 10.9 million cases and 116,800 deaths per year [1,2]. Infections follow after ingestion of contaminated food or water [3] and are predominantly confined to low- and middle-income countries (LMICs) in Asia and sub-Saharan Africa (sSA) [4]. In 2004, the burden of typhoid fever in sSA was estimated at 10–100 cases per 100 000 per year [5], while more recent surveillance data estimated the overall adjusted incidence rate to be more than 100 per 100,000 in children younger than 15 years [4].

Since the 1970s, *S*. Typhi has increasingly exhibited multidrug resistance (MDR), defined as co-resistance to the traditional first-line antibiotics ampicillin, chloramphenicol, and sulfamethoxazole-trimethoprim (co-trimoxazole) [6–9]. A shift in treatment to fluoroquinolone antibiotics was subsequently followed by decreased ciprofloxacin susceptibility (DCS) [10,11] and treatment failure [11,12]. For inpatients, the currently recommended antibiotic to treat typhoid fever next to ciprofloxacin is the cephalosporin class antibiotic ceftriaxone [13]. Recently cephalosporin resistance due to the production of extended spectrum β-lactamases (ESBL) by *S*. Typhi underlied a large outbreak in Pakistan [14,15], while reports in sSA have been sporadic [16,17]. Azithromycin remains an option for typhoid treatment, although the World Health Organization (WHO) has called for vigilance concerning the emergence of resistance to this antibiotic [18].

The introduction of whole genome sequencing (WGS) has yielded insights in the genetic diversity of *S*. Typhi, thereby showing dominance of clonal haplotype H58 (genotype 4.3.1) [19]. This haplotype showed intercontinental transmission from South Asia to East Africa and is associated with high levels of MDR and DCS [20]. While genotype 4.3.1 is dominant in East sSA [21,22], genotype 3.1.1 dominates West sSA [21].

The genetic diversity and AMR of *S*. Typhi in Rwanda remains largely unknown. In the 1980s, three studies on *S*. Typhi isolates were performed in Butare and Kigali showing full susceptibility to all tested antibiotics for all isolates [23–25]. A later study conducted at King Faisal Hospital in Kigali reported an increase of phenotypic MDR from 9.1% (3/33) in 2007 to 25% (9/36) in 2008 [26].

WGS generates high-resolution genetic information, which is valuable for surveillance of bacterial outbreaks and AMR [27,28]. In some high- income settings, it has replaced the classical molecular typing methods to reveal genotype, AMR markers and genetic relationships among bacterial isolates [29,30].

We postulate that WGS is a highly valuable methodology in low-income settings and can be implemented to support bacterial surveillance work. Therefore, in this study, we analyzed the genomes of 51 *S*. Typhi, including historical (1984-1985) and recent (2010-2018) isolates from Rwanda, for the presence of AMR markers and genetic relationship within a global *S*. Typhi phylogeny. In addition, we provide a workflow to conduct WGS at national reference laboratories in typhoid fever endemic countries and discuss the opportunities and pitfalls for successful implementation.

## Materials and methods

### Bacterial isolates

Fifty-one *S*. Typhi isolates were isolated from blood samples collected as part of research studies in Rwanda and were included in this study. Twenty-five isolates were part of a historical collection stored at the Saint Pierre University Hospital in Brussels (CHU Saint-Pierre), Belgium and these were isolated in 1984 and 1985 from 3 districts in Rwanda. Twenty-six isolates from a more recent collection were isolated during research studies by the Rwandan National Reference Laboratories (NRL) and during clinical care at the Centre Hospitalier Universitaire de Kigali (CHUK) between 2010 and 2018 covering 17 out of 30 districts of Rwanda. Year and location of isolation for each isolate are presented in S1 Table.

### Serotyping and antibiotic susceptibility testing (AST)

The 25 historical isolates were retrieved from -80°C storage at CHU Saint-Pierre in Belgium, subcultured and shipped to the Institute of Tropical Medicine (ITM) in Antwerp, Belgium for serotyping and antibiotic susceptibility testing (AST). The 26 recent isolates were retrieved from -80°C at NRL and CHUK in Kigali, Rwanda, regrown and subjected to serotyping and AST at the NRL. All isolates were serotyped using commercial antisera (Pro-Lab Diagnostics, Richmond Hill, Canada) following the Kauffmann-White-Le-Minor scheme [31]. The AST was determined by disc diffusion [Neo-Sensitabs, Rosco Diagnostica, Taastrup, Denmark] and E-test macromethod [bioMérieux, Marcy-l’Étoile, France] for determination of the minimal inhibitory concentration (MIC) for azithromycin and ciprofloxacin on Mueller-Hinton agar following the Clinical and Laboratory Standards Institute (CLSI) guidelines of 2018 [32]. For the historical isolates, the following antimicrobial drugs were tested: cotrimoxazole, gentamicin, tetracycline, pefloxacin, chloramphenicol, nalidixic acid, ertapenem, meropenem, ceftriaxone, ampicillin, ceftazidime, azithromycin and ciprofloxacin. For the recent isolates, the same panel of antimicrobial drugs was tested except nalidixic acid and ertapenem, but it also additionally included imipenem. DCS was defined as intermediate ciprofloxacin resistance (MIC between 0.064 μg/ml and 1 μg/ml). Isolates were assessed for possible production of extended spectrum β-lactamases (ESBL) by the double disk test with ceftazidime (CAZ30) in combination with ceftazidime and clavulanic acid (CAZ30+C), and cefotaxime (CTX30) in combination with cefotaxime and clavulanic acid (CTX30+C). In case a difference of ≥ 5 mm is observed in either ceftazidime or cefotaxime clavulanic acid inhibition tests the ESBL production is considered.

### DNA extraction

DNA was extracted from overnight *in vitro* cultures in LB broth (miller) (Merk, Darmstadt, Germany) at 37°C. Total DNA was extracted from bacterial cell pellets using the Gentra Puregene Yeast/Bact. kit (Qiagen, Hilden, Germany) according to the manufacturer’s instructions. Total DNA was eluted in 50 μL DNA hydration solution and concentrations were measured with Qubit 4 Fluorometer using the Qubit dsDNA High Sensitivity Assay Kit (Thermo Fischer Scientific).

### Illumina sequencing

Historical isolates were whole genome sequenced at ITM and the recent isolates at NRL. For both runs, indexed paired-end libraries were prepared using the Nextera XT DNA Library Prep Kit V2 Set B (Illumina, San Diego, California, USA) from 1 ng DNA input per sample. Libraries of the historical isolates were quantified with Qubit™ dsDNA HS assay kit (Invitrogen, Carlsbad, California, USA) at >10-15 nM and were normalized using the bead-based method and pooled. For the recent isolates, the Qubit™ dsDNA HS assay kit quantification of individual libraries resulted in concentrations of less than 10 nM and were therefore normalized using the standard normalization method at 1 nM and pooled. Pooled libraries in both runs were quantified with the Kapa Library Quantification Kit (Roche Applied Science, Penzberg, Germany) and were subsequently denatured and diluted to the loading concentration of 1,8 pM. Five hundred μl of the 1,8 pM libraries were combined with 5 μl of denatured PhiX control at 1,8 pM and the total volume was loaded onto MiniSeq System High-Output cartridge for sequencing on the Illumina MiniSeq instrument (Illumina, San Diego, California, USA).

### Quality assessment of sequence data and assembly

Raw reads were analysed using FastQC (v0.11.9) and MultiQC (v1.8), prior to trimming with Trimmomatic (v0.39) [33]. Trimmed reads were used in the following steps for further quality assessment: taxonomic read classification was performed with kraken2 v2.0.8_beta [34], to confirm that the species-level taxon to which kraken2 assigned the highest number of reads was *Salmonella enterica* and not a contaminant. The estimated sequencing depth, *i*.*e*. the estimated number of times a base was sequenced, was calculated by dividing the number of sequenced bases by the genome size.

Reads were mapped to the *S*. Typhi strain CT18 reference genome (accession number NC_003198.1) using minimap2 (options: -ax sr) [35], and further processed using samtools v1.10 [36]. Genome coverage was assessed by counting the number of genome positions that was covered by at least one sequencing read from the output of samtools depth (options: -aa -q 20 -Q 60). The percentage of reads mapping to the reference genome was calculated with the samtools flagstat utility. Trimmed reads were de novo assembled using spades v3.14.0 (options: --careful --only-assembler) [37].

Quality metrics such as number of contigs, largest contig, total length, GC (%), N50 and NGA50 were calculated using quast v5.0.2 [36]. The number of coding sequences was inspected per sample after annotation with Prokka v1.14.6 [38], and sistr v1.1.0 [39] was used to confirm the serotype *in silico*.

### Web-based analysis of sequence data: prediction of serovar, genotypes, and AMR genetic determinants

The genome assemblies and their corresponding metadata in csv format (file name, display name, geographical coordinates and year of isolation) [S2 Table] were uploaded to the open-source online Pathogenwatch tool [v3.11.9; April 2020] developed by the Centre for Genomic Pathogen Surveillance at the Wellcome Trust Sanger Institute (UK) [https://pathogen.watch]. Pathogenwatch allows fast predictions of resistant genotypes and clustering among isolates by generating phylogenetic trees (https://cgps.gitbook.io/pathogenwatch/technical-descriptions/core-genome-tree/tree-construction). This is based on classifying to closest references and then adding the isolates to the tree using a neighbor joining algorithm.

Serovar identification, multi-locus sequence typing (MLST), plasmid incompatibility (inc) type and genotypes were determined per isolate as well as AMR genetic determinants for ampicillin, cephalosporins, chloramphenicol, ciprofloxacin, nalidixic acid, sulfamethoxazole, trimethoprim, co-trimoxazole, tetracycline, azithromycin, colistin and meropenem.

The Pathogenwatch predicted AMR genetic determinants were complemented with an analysis in ARIBA software (v.2.14.6) [40] and CARD database (3.0.9) [41], for further comprehensiveness.

A phylogenetic tree showing the genetic relationship among all 51 isolates was generated based on the analysis of all genome sequences in Pathogenwatch. The obtained phylogenetic tree was subsequently uploaded as nwk file into Microreact [V5.93.0; April 2019] together with a csv file with metadata [S3 Table] for interactive visualization. Microreact complements to Pathogenwatch by providing a quick interactive visualization of phylogenetic trees in contrast to the static trees often generated by other different online tools [42].

### Bioinformatic analysis: high-resolution phylogeny

A high-resolution phylogenetic analysis of the identified dominant *S*. Typhi H58 haplotype was conducted in the context of global *S*. Typhi population. Hereto, Illumina MiniSeq reads were mapped to the *S*. Typhi reference genome CT18 (NC_003198.1) with SMALT v0.7.4 and indexed using a kmer size of 20 and a step size of 13. PCR duplicate reads were identified using Picard v1.92 (Broad Institute, Cambridge, MA, USA) and flagged as duplicates. Variants were detected using Samtools Mpileup v0.1.19 with parameters “-d 1000 -DSugBf” and bcftools v0.1.19 [43]. The bcftools variant quality score was set to be larger than 50 and mapping quality greater than 30. The allele frequency was set to be larger than 95%. The majority base call was set to be present in at least 75% of reads mapping at the base, and the minimum mapping depth was 4 reads, at least two of which had to map to each strand. Finally, strand bias was set to be less than 0.001, map_bias less than 0.001 and tail_bias less than 0.001.

A pseudo-genome was constructed by substituting the base call at each site in the reference genome and uncertain sites were substituted with an N. Deletions with respect to the reference genome were filled with N’s in the pseudo-genome to keep it aligned. Prophage regions in the chromosome were identified using Phaster [44] and removed from the chromosome alignment. SNP sites were extracted from the alignment using snp-sites [45] and used to construct a maximum likelihood phylogeny with RAxML [46] (v8.2.8) with substitution model GTRGAMMA.

A phylogenetic analysis for genotype 4.3.1 was conducted on 22 isolates from our study combined with 915 isolates retrieved from a previous publication [19], based on 2091 SNPs. Support for nodes on the phylogenetic trees assessed using 1000 bootstraps; and the tree was outgroup-rooted on one non 4.3.1.2 *S*. Typhi isolate from Fiji, (accession number ERR357459). The generated phylogenetic trees were then subsequently visualized with iTOL [47]. Metadata of all isolates used for visualization in iTOL are listed in S4 and S5 Tables.

## Results

### Implementation of *S*. Typhi WGS

**Fig 1:**
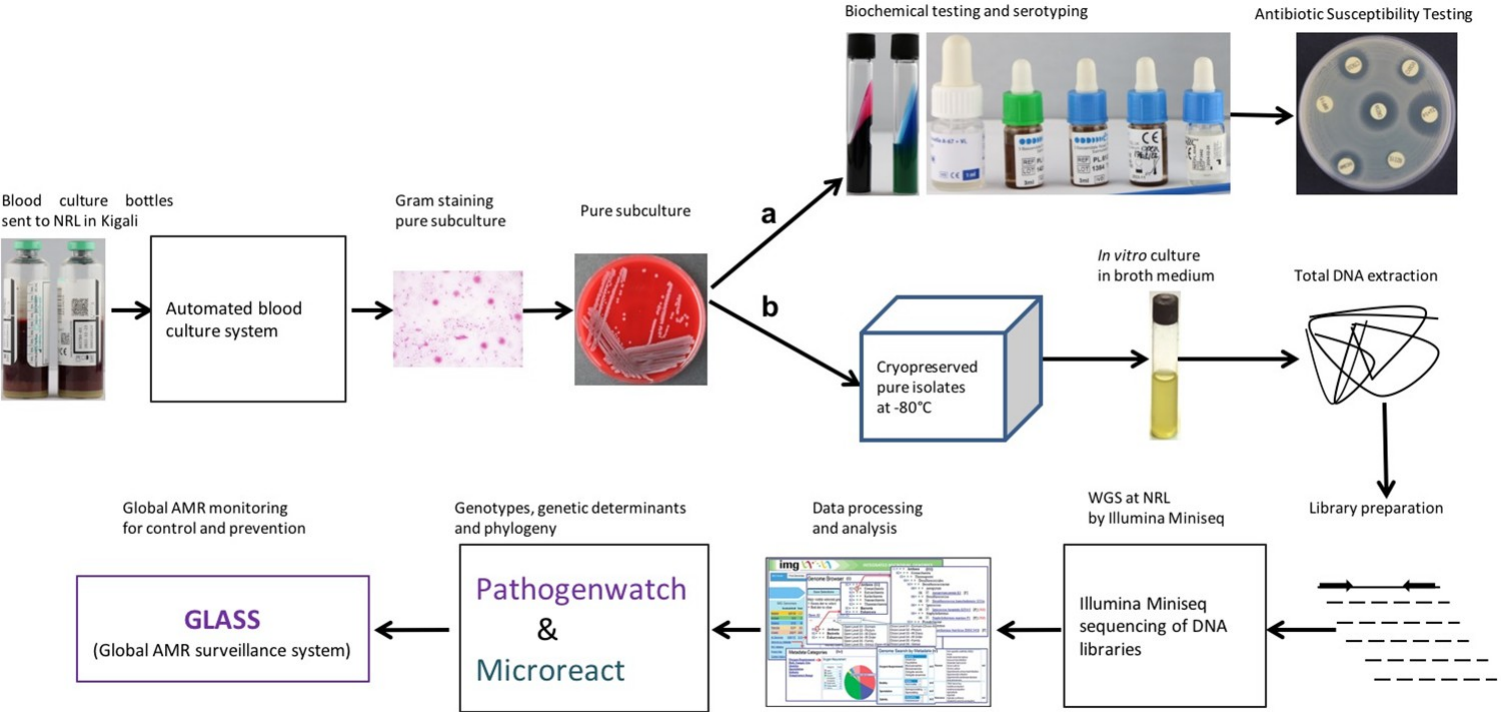
Workflow for implementation of WGS in typhoid surveillance. Microbiological work-up of bacterial isolates, which includes bacterial identification (Gram staining, pure subculture and serotyping) and antimicrobial susceptibility testing (AST) (a); A WGS workflow, starting with cryopreservation of pure *S*. Typhi isolates, including determination of genotypes and genetic determinants of AMR, may result in sharing data in the global AMR monitoring for control and prevention (GLASS) programme (b).

*S*. Typhi WGS using the Illumina MiniSeq sequencer was implemented in parallel at NRL in Rwanda and ITM in Belgium, and the used workflow is presented in Fig 1. The routine diagnostic workflow for *S*. Typhi bloodstream infection started with blood culture, followed by Gram staining, biochemical testing, *Salmonella* serotyping, and AST [Fig 1A]. The WGS workflow starts with stored, pure *S*. Typhi isolates from blood cultures -80°C followed by *in vitro* cultures in LB broth. For WGS analysis total DNA was extracted, followed by DNA library preparation and Illumina MiniSeq sequencing. Analysis of processed sequence data allowed to determine genotypes, AMR genetic determinants and phylogenetic relationship among isolates [Fig 1B]. The WGS workflow thus complements the available routine diagnostic workflow with additional information.

Quality control of WGS data confirmed all isolates in the historical and recent collections as *S*. Typhi. Less than 0.1% of the reads were classified as a species other than *Salmonella enterica* by kraken2, and therefore no contamination was detected. Genome coverage was consistently >96%, but with eight isolates showing relative low depth (S6 Table): 438_85, 2688_CHUK, 270_13, 380_13, 61_15, B014_11, B103_10 and BS055_10, which resulted in a lower average sequencing depth for the recent isolates [Tables 1, S5 and S6]. Despite the lower sequencing depth recorded for these isolates, we retrieved enough information to construct the maximum likelihood phylogenetic tree and to predict gene presences/absences. WGS quality statistics are presented in Tables 1 and S6.

**Table 1:** Summary of the sequencing parameters [mean, median and standard deviation of the average sequencing depth, percentage of coverage, percentage of GC content, and number of coding sequences (CDS)] of the two MiniSeq runs: historical isolates and recent isolates sequenced at ITM and NRL, respectively. Average sequencing depth is the number of sequenced bases divided by the genome size; coverage (%) is the percentage of the genome that is covered by at least one (well-mapped) read; GC (%) is the percentage of G and C nucleotides, CDS is the number of genes (coding sequences), and SD is the standard deviation.

	Historical isolates				Recent isolates			
	Average sequencing depth	Coverage (%)	GC (%)	CDS	Average sequencing depth	Coverage (%)	GC (%)	CDS
Mean	55	97,13	52	4530	41	97,23	52	4679
Median	52	97,06	52	4514	28	97,29	52	4648
Standard deviation (SD)	23	0,2	0	34	35	0,23	0	100

During implementation of WGS at the NRL in Rwanda, the five practical challenges we identified were: high cost of shipment and customs for expensive sequencing reagents to Rwanda, the lack of systematic storage during routine diagnostic work-up and inventory of isolates in the -80°C freezer, limited access to high end bioinformatic facilities and data storage, and the lack of connection between laboratory registries and laboratory information system (LIS).

### AMR in *S*. Typhi increased over time in the samples from Rwanda

WGS data were analyzed with the web-based tool Pathogenwatch [48] to provide AMR genetic determinants and plasmid Inc types. None of the historical isolates showed resistance markers underlying MDR (resistance to ampicillin, chloramphenicol and sulfamethoxazole-trimethoprim) or DCS (decreased susceptibility to the fluoroquinolone ciprofloxacin), and these were thus observed to be susceptible to all treatment options [S1A Fig]. None of the historical and recent isolates presented ESBL production nor azithromycin resistance markers (S7 Table).

The majority of the recent isolates however presented markers for DCS (22/26; 84.6%) of which all but one showed MDR markers (21/26; 80.8%) [Tables 2 and S7 and S1 Fig]. All genetic AMR results matched the phenotypic AST determinations [S7 and S8 Tables].

**Table 2:** AST profiles and associated genetic markers among recent (2010-2018) isolates.

Strain ID	SxT		CHL		AMP		CIP	
	AST	Genetic markers	AST	Genetic markers	AST	Genetic markers	AST	Genetic markers
B033_10, B052_10 and B069_10	R	*sul1*, *sul2* & *dfrA7*	R	*catA1*	R	*blaTEM-1D*	I	*gyrA_S83Y*
B091_10	I		S		S		S	
B098_10	R	*sul1* & *dfrA7*	R	*catA1*	R	*blaTEM-1D*	I	*gyrA_S83Y*
B103_10, BS026_10, 380_13 and 61_15	R	*dfrA7*	R	*catA1*	R	*blaTEM-1D*	I	*gyrA_S83Y*
B105_10	S		S		S		I	*gyrA_S83Y*
BS042_10, BS055_10 and BS056_10	R	*sul1*, *sul2* & *dfrA1*	S		R	*blaTEM-1D*	S	
B009_11, 253A_13, 326A_13, 474_13, 414_14, 2404_CHUK, 2741_CHUK, 2970_CHUK and 2929H_CHUK	R	*sul1*, *sul2* & *dfrA7*	R	*catA1*	R	*blaTEM-1D*	I	*gyrA_S83Y*
B014_11	R		R	*catA1*	R	*blaTEM-1D*	I	*gyrA_S83Y*
270_13	R	*sul2* & *dfrA7*	R		R	*blaTEM-1D*	I	*gyrA_S83Y*
514_15 and 2688_CHUK	R	*sul2* & *dfrA7*	R	*catA1*	R	*blaTEM-1D*	I	*gyrA_S83Y*

R= resistant; S= susceptible; SXT= sulfamethoxazole-trimethoprim (co-trimoxazole); CHL= chloramphenicol; AMP= ampicillin and CIP= ciprofloxacin.

The identified MDR genes were *sul1* and *sul2* (sulfamethoxazole, cotrimoxazole), *dfrA1* and *dfrA7* (trimethoprim, cotrimoxazole), *catA1* (chloramphenicol) and *blaTEM*-1D (ampicillin). The predicted AMR genetic determinants with Pathogenwatch were also complemented with analysis in ARIBA: the latter confirmed the observed genotype-phenotype matches and mismatches [Tables 2 and S9].

Almost half (42,9%, 9/21) of MDR isolates were associated with the presence of an IncHI1 replicon while no plasmid replicon was identified in 47,6% (10/21) [S7 Table], suggesting possible chromosomal insertion of the MDR genes. All 22 genotype 4.3.1.2 recent isolates exhibiting DCS, presented a S83Y substitution in the *gyrA* gene.

### A diverse historical population has recently been replaced by H58

**Fig 2:**
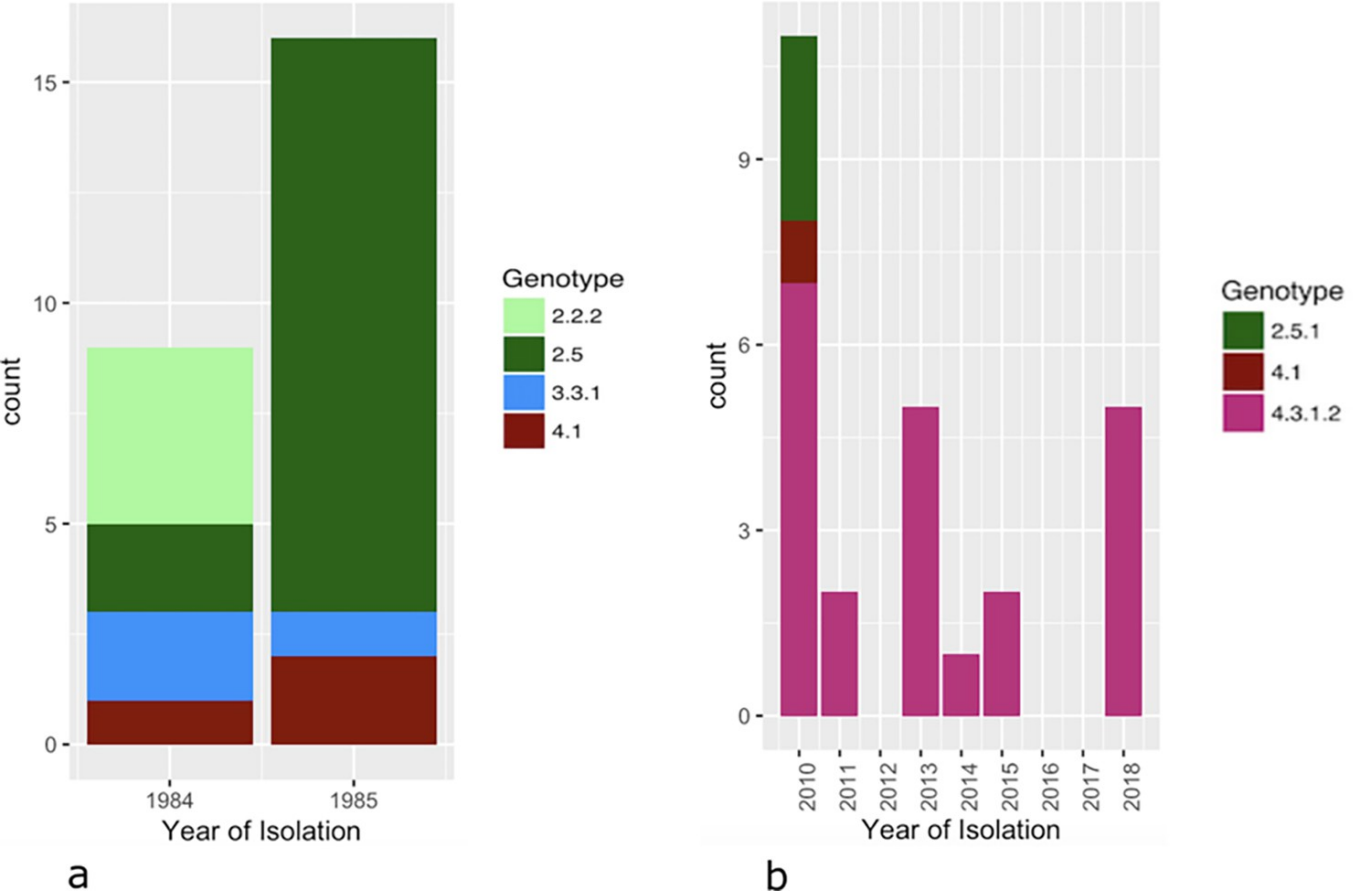
Distribution of *S*. Typhi genotypes among historical (a) and recent (b) *S*. Typhi isolates per year of isolation.

**Fig 3:**
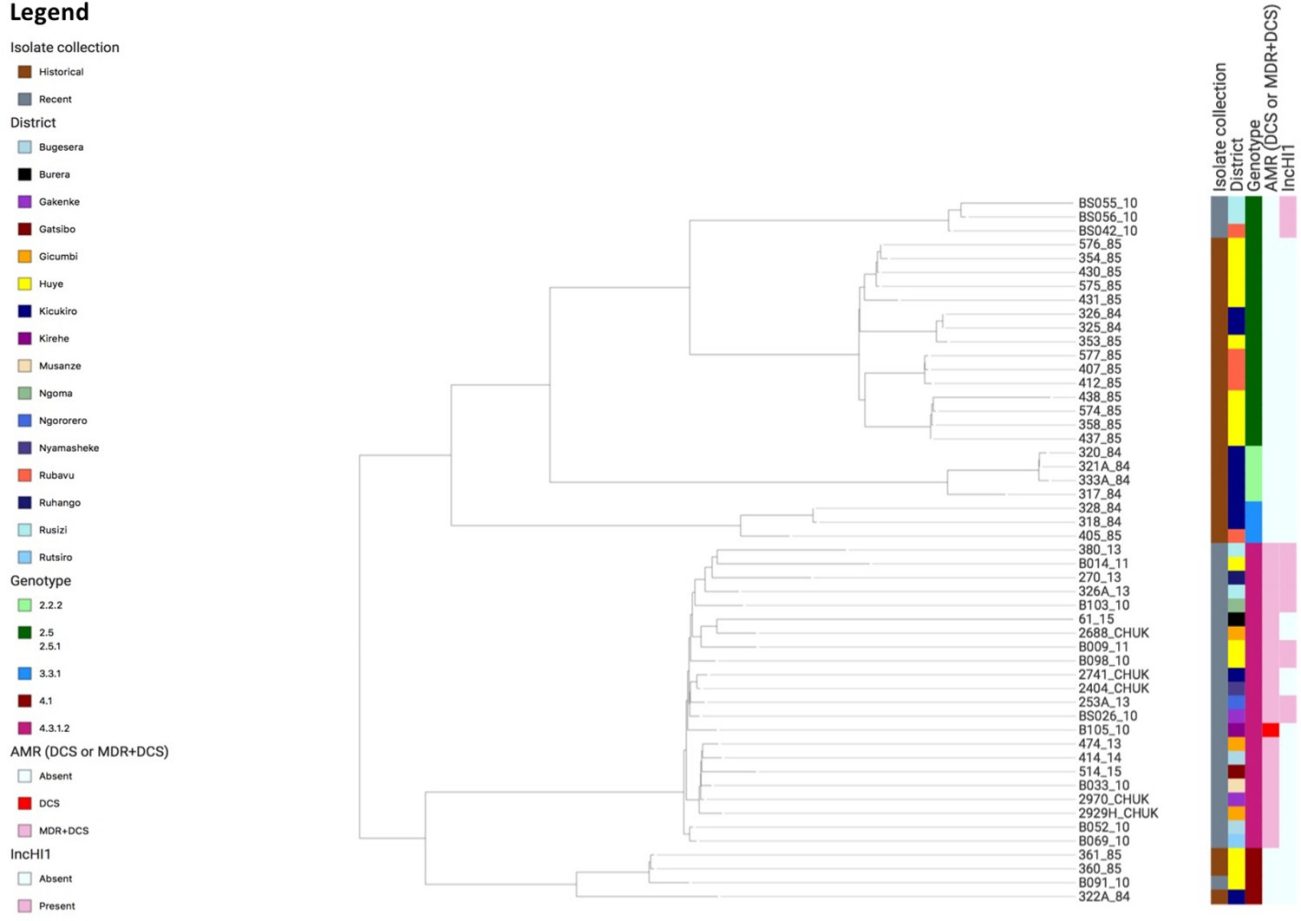
Phylogenetic tree of the *S*. Typhi isolates as generated in Pathogenwatch and visualized in Microreact (https://microreact.org/project/syLwGwmBRvbSbVeq1sET2x). Metadata defined in the legend indicate, from left to right (i) the type of isolate collection (recent or historical), (ii) district, (iii) genotype, (iv) AMR [multidrug resistant (MDR), decreased ciprofloxacin susceptibility (DCS), or MDR + DCS), (v) presence or absence of the IncHI1 plasmid replicon per isolate.

Among the historical *S*. Typhi isolates, four different genotypes were observed: 2.5 was most dominant (60%, 15/25), followed by 2.2.2 (16%, 4/25), 3.3.1 and 4.1 (both 12%, 3/25) [Fig 2A] [S7 Table]. A diverse population was associated with both surveillance years and with the different locations of surveillance (Fig 3).

In contrast, the recent isolates were dominated by genotype 4.3.1.2 (H58, 22/26; 84,6%), followed by 2.5.1 (3/26; 11,5%) and 4.1 (1/26; 3,8%). While genotype 4.3.1.2 coexisted with genotypes 2.5.1 and 4.1 in 2010, it was the sole identified genotype from 2011 onwards. Interestingly, genotype 4.1 was found in both historical and recent *S*. Typhi isolates, suggesting a possible long-term persistence of this genotype in the region.

A population analysis of the *S*. Typhi isolates and associated metadata as presented by Pathogenwatch (Fig 3) showed that MDR and DCS were restricted to the 4.3.1.2 branch and were not found in genotypes 2.5.1 and 4.1. Genotype 4.3.1.2 isolates were found across the country in different districts, while the other genotypes were often restricted to specific regions, for example with genotype 2.2.2 only found in Kicukiro and genotype 2.5 predominantly found in Huye [Fig 3 and https://microreact.org/project/syLwGwmBRvbSbVeq1sET2x].

### A distinct introduction of genotype 4.3.1.2 (H58) in Rwanda

**Fig 4:**
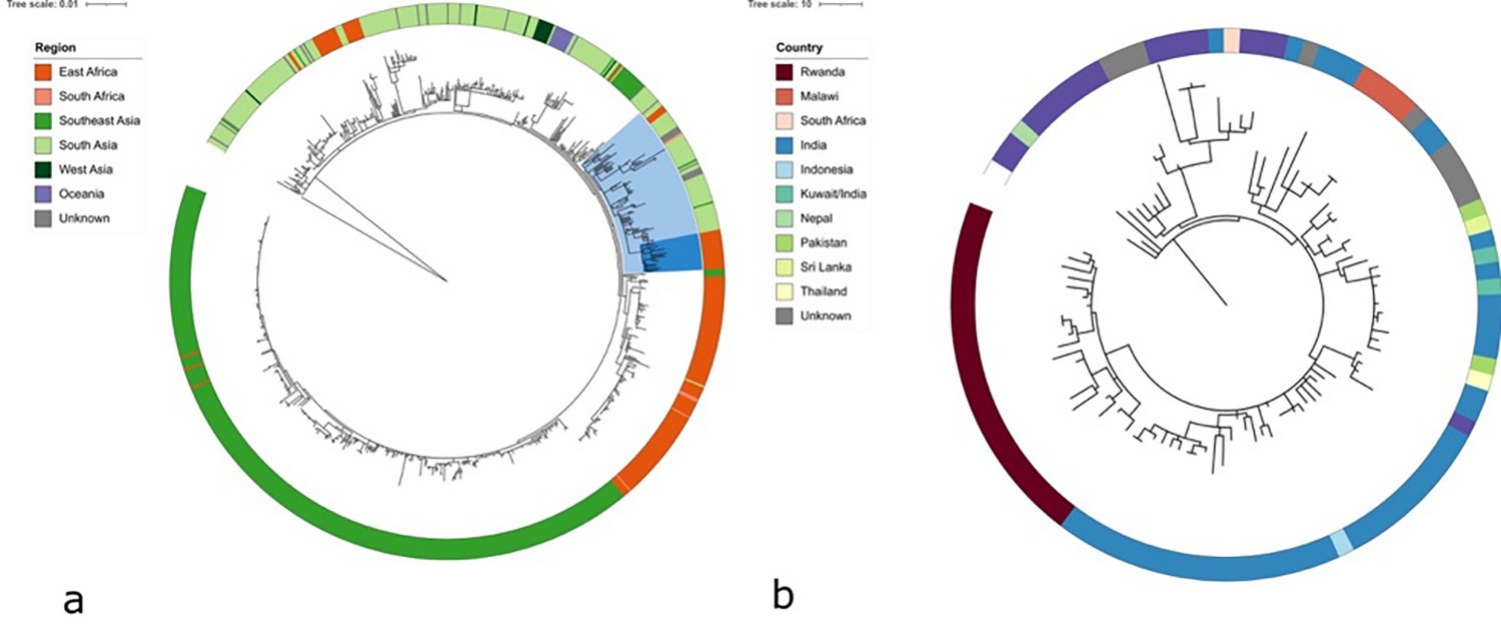
Maximum likelihood phylogenetic tree of 22 genotype 4.3.1.2 isolates from Rwanda in the context of global genotype 4.3.1 *S*. Typhi (H58) isolates (a); and a detailed phylogenetic tree in the context of neighbor isolates (b).

The 22 genotype 4.3.1.2 (H58) genomes from Rwanda were further analyzed in global 4.3.1 context [19] using in depth-bioinformatic approaches and a maximum likelihood phylogeny was constructed as described under the section of materials and methods above, and were found to form a monophyletic cluster within the 4.3.1 phylogeny [Fig 4A]. Our data suggest a separate introduction and subsequent local expansion of the genotype in Rwanda. The majority of isolates neighbouring the isolates from Rwanda originate from South Asia, and more specifically India. Similarly, to the monophyletic branch of isolates from Rwanda, a separate monophyletic branch with related isolates originate from Malawi [Fig 4B]. There is therefore no evidence for the introduction of local H58 from East or South Africa, but conclusions remain speculative, given that data from this region is intrinsically scarce. There is an important sample bias and limited detailed information for many endemic sites.

The phylogeny was inferred from 2091 SNPs; rooted using an outgroup of a non-H58 related strain, genotype 4.2.0 *S*. Typhi isolate from Fiji (accession number ERR357459). Branch lengths are indicative of the estimated substitution rate per variable site. Colored rings indicate the region of isolation (panel a), and country of origin (panel b). Countries are classified by region following the UN geoscheme. In panel a, the dark blue range indicates the isolates from Rwanda, the bright blue range indicates the subselection with neighbor isolates analysed in panel B. The used metadata for panel A and B are presented in S4 and S5 Tables, respectively.

## Discussion

We present a genomics perspective of 51 *S*. Typhi isolates originating from two different time periods (historical and recent) in Rwanda. Sequencing was conducted at NRL Rwanda and ITM Belgium using a MiniSeq Illumina platform, a benchtop and user-friendly sequencer, which is also the second most affordable and the least time-consuming Illumina platform. Despite the faced challenges, the implemented WGS workflow was successful in our setting. Our workflow incorporated user-friendly and web-based analysis tools. These online tools increase accessibility of WGS analysis at a low cost and with limited need for specialized expertise. On the other hand, such web-based tools did not provide the same flexibility and resolution as in depth bioinformatic methods and they were also not specifically approved for clinical decision making as the phenotypic assays, which would be an interesting evolution for the future.

WGS results yielded the same accuracy for AMR surveillance as compared to classical laboratory methods, and gives additional information on genotype, population structure and plasmid presence. WHO recently published a technical note to assist countries in the implementation of WGS for AMR surveillance and support sharing WGS data in GLASS, which can provide early information on the spread of AMR pathogens at the local, regional and international level and allows for timely devised policies and interventions to control AMR [28].

Implementation of WGS to routine bacterial surveillance has large potential for revealing crucial information on emerging clonal haplotypes. WGS will remain complementary to phenotypic characterization because genotype-phenotype linkage is not always complete [49]. In Rwanda, applying WGS at the NRL will help to improve or confirm outbreak detection, and add genetic resolution to bacterial isolates and their AMR. In case of typhoid fever outbreaks in the country, WGS can help to assess whether it is a result of a novel introduction or an endemicity of Typhi strains. Additionally, these first WGS data of *S*. Typhi in Rwanda provide baseline data on the local bacterial population structure, which might be addressed during future intervention studies.

In Rwanda, the historical *S*. Typhi population was more diverse as compared to the recent population which was dominated by genotype 4.3.1.2 (lineage H58) and presents MDR and DCS. In line with this, a genotype 4.3.1.2 isolate was recently identified at the Public Health England from a traveller returning from Rwanda [50].

Our study presents substantial geographical distribution of the recent genotype 4.3.1.2 isolates in the country which is not observed for the older genotypes, show a lower level of dispersal at the country-level. This might be due to differences of *S*. Typhi transmission per genotype, or to migration of people in the different time periods, but there might also be sampling bias. Our data also suggest a population replacement of the historical genotypes by H58; similar to what has previously been observed in different countries of Asia and sSA [51–53]. Genotype 4.3.1 has emerged in Asia and has shown intercontinental transmission from South Asia to East Africa where it established [20,22,51,54]. Our data suggest a separate introduction in Rwanda giving rise to a stable population, possibly directly from Asia. We observed no obvious evidence for further transmission between Rwanda and other African countries. However, due to sample sparsity in the region, there might be a sampling bias underlying these findings.

With the introduction of 4.3.1.2 in Rwanda, *S*. Typhi infections showed MDR and DCS. Remaining treatment options are the third-generation cephalosporin ceftriaxone and the macrolide azithromycin. Within Africa, AMR is especially high among *S*. Typhi within the East African region [9,55,56], although MDR and DCS have also been reported in Central [57] and West Africa [58–60].

In contrast to the current dominance of *S*. Typhi haplotype 4.3.1, a more diverse historical *S*. Typhi population was likely present historically in Rwanda. Based on the publicly available WGS of isolates from before the 1990s this might also be true elsewhere in the world; *S*. Typhi from Asia (Vietnam, Indonesia and India) were assigned to genotypes 2.1.7, 2.5.0, 3.2.1, 3.4.0 and 4.1.0 and from Africa (Cameroun, Tunisia, the Democratic Republic of the Congo (DRC) and Madagascar) to 0.1.1, 1.1.3, 2.2.0, 2.3.1 and 3.1.0 [19]. Although these historical data are sparse and limited, it suggests a higher diversity of genotypes among these historical isolates compared to recent ones from these regions.

The main limitation to the study is sample scarcity. Our sample set is opportunity-driven and therefore likely biased. A countrywide standardized blood culture surveillance [61] coupled to WGS would be needed to provide a systematic view on the incidence and AMR of typhoid fever in Rwanda, which could guide the introduction of typhoid vaccination programmes in the country. Rwanda is eligible for the support from Gavi for the introduction of new vaccines such as typhoid conjugate vaccines (TCVs). Two Vi-TCV, Typbar-TCV and TyphiBEV, have been prequalified by WHO in 2017 and 2020, respectively [62]; and they have shown safety and efficacy in Malawi [63] and Bangladesh [64].

## Supporting information

S1 Fig Distribution of genetic MDR and DCS markers. (JPG)

S1 Table Historical and recent isolates. (XLSX)

S2 Table Pathogenwatch metadata. (CSV)

S3 Table Microreact metadata. (CSV)

S4 Table iTOL_Genotype 4.3.1.2, datasets_region. (XLSX)

S5 Table iTOL_Genotype 4.3.1.2_ Rwanda_metadata. (XLSX)

S6 Table Quality metrics and statistics of sequences. (XLSX)

S7 Table AST & genetic determinants. (XLSX)

S8 Table AST results. (XLSX)

S9 Table Analysis_AMR_ARIBA. (XLSX)
